# Efficient Low-PAR Waveform Design Method for Extended Target Estimation Based on Information Theory in Cognitive Radar

**DOI:** 10.3390/e21030261

**Published:** 2019-03-07

**Authors:** Tianduo Hao, Chen Cui, Yang Gong

**Affiliations:** National University of Defense Technology, Hefei 230037, China

**Keywords:** waveform design, mutual information (MI), peak-to-average power ratio, minorization–maximization (MM) method, cognitive radar

## Abstract

This paper addresses the waveform design problem of cognitive radar for extended target estimation in the presence of signal-dependent clutter, subject to a peak-to-average power ratio (PAR) constraint. Owing to this kind of constraint and the convolution operation of the waveform in the time domain, the formulated optimization problem for maximizing the mutual information (MI) between the target and the received signal is a complex non-convex problem. To this end, an efficient waveform design method based on minimization–maximization (MM) technique is proposed. First, by using the MM approach, the original non-convex problem is converted to a convex problem concerning the matrix variable. Then a trick is used for replacing the matrix variable with the vector variable by utilizing the properties of the Toeplitz matrix. Based on this, the optimization problem can be solved efficiently combined with the nearest neighbor method. Finally, an acceleration scheme is used to improve the convergence speed of the proposed method. The simulation results illustrate that the proposed method is superior to the existing methods in terms of estimation performance when designing the constrained waveform.

## 1. Introduction

Cognitive radar (CR) is a new intelligent closed-loop radar system that can perceive the surrounding complicated electromagnetism environment in real time and make reasoning decisions on this basis [1,2]. In CR, adaptive transmitted waveform design based on the perceived prior knowledge of environment and target is one of the key technologies which can significantly improve the performance of target detection, parameter estimation, recognition, and tracking in complicated environments [3].

For each of these missions, there are corresponding valid waveform design methods [4]. When designing waveform for target estimation, minimum mean squared error (MMSE) criterion [5], the minimum Cramer-Rao lower bound (CRLB) criterion [6,7] and maximum mutual information (MI) [5,8,9] criterion are usually selected. However, the CRLB criterion is only suitable for situations in which the target information is unknown. Meanwhile, maximization of MI and the minimization of the MMSE lead to the same solution when the target information is known.

The authors in [10] proposed an estimation waveform design method based on MI in noise, in which the original non-convex problem was converted to a convex problem by using the convex optimization method. Considering the signal-dependent clutter, the waveform design methods for target estimation based on MI and MMSE were proposed in [11,12], respectively. It is worth noting that the envelope constraint on the transmitted waveform was not considered in any of those studies, which made it difficult to meet the hardware constraints and maximize the power efficiency [13]. For this reason, unimodular or low peak-to-average power ratio (PAR) waveform is always applied in radar systems [13,14]. Nevertheless, unimodular waveform may lead to the degradation of waveform performance [15]. To tackle this problem, some researchers had used a more general low-PAR constraint to replace the unimodular constraint to further improve the waveform performance [6,7,14,15,16,17,18].

In [6], subject to the constraint, a frequency domain based PAR waveform design method with the MI criterion was proposed. However, the algorithm in [6] can only find the envelope of optimal waveform spectrum. Since the spectral phase cannot be determined, the number of time domain signals that satisfy the unique waveform spectrum magnitude is infinite. Therefore, it may result in a decline of the waveform performance when the frequency domain waveform spectrum is transformed to the time domain waveform [19]. To solve this problem, an algorithm based on the sequence linear programming (SLP) in time domain was proposed in [20], and the original non-convex problem was converted a convex problem which could be solved efficiently. It is worth noting that the optimization problem in [20] is the relaxation of the original problem so the synthesized waveform may be still the suboptimal solution.

As is well known, the minorization–maximization (MM) method is a powerful optimization technique to solve the hard problem that is difficult to tackle directly [21]. The core principle of MM is to transform the original problem into a series of simple problems which can be tackled efficiently and converge to the stationary optimal solution of the original problem [22]. Motivated by the ascent property and superior convergence of MM, it has been applied in many fields [23,24,25,26,27].

In this paper, we propose an efficient low-PAR cognitive waveform design method based on the MM approach for target estimation, which is directly studied in the time domain. Based on the MM approach, the original non-convex problem is converted to a convex problem with respect to (w.r.t.) a matrix variable. To reduce computation cost, the convex problem is further converted to quadratic programming (QP) problem w.r.t. a vector variable by utilizing the properties of the Toeplitz matrix. Based on this, the QP problem is converted to a simple convex problem which can be tackled efficiently by using the nearest neighbor method. Finally, the squared iterative methods (SQUAREM) is used to improve the convergence speed of the proposed method. The simulation results demonstrate that the synthesized waveform can be obtained efficiently within the given low-PAR range and the proposed method has better estimation performance than the existing methods.

The remainder of the paper is organized as follows: Section 2 gives the baseband radar signal model. In Section 3, the optimal criterion based MI is formulated, and an efficient low-PAR waveform design method based on MM is proposed. A detailed performance analysis of the proposed method is provided in Section 4. Section 5 presents our simulation results. Finally, the conclusion is summarized in Section 6.

*Notation*: Scalars are represented by italic letters, vectors and matrices are denoted by boldface lowercase and uppercase letters, respectively. The superscripts in ${\left(\cdot \right)}^{T}$ and ${\left(\cdot \right)}^{H}$ represent the transpose and Hermitian transpose operations, respectively. A(m,n) denotes the element located in the mth row and nth column of A.F(·) denotes the Toeplitz matrix mapping function of a vector, ℜ(·), ℑ(·), | · | and ‖ · ‖ represent the real part, imaginary part, modulus and 2-norm of a complex scalar/vector/matrix, respectively. ℂ is the set of complex-valued number. The symbol ‘⊗’ and ‘*’ denote the Kronecker product and the convolution operation, respectively. Finally, CN(0,A) denotes a circular symmetric complex Gaussian distribution with zero mean and the covariance matrix A.

## 2. Signal Model

**Figure 1:**
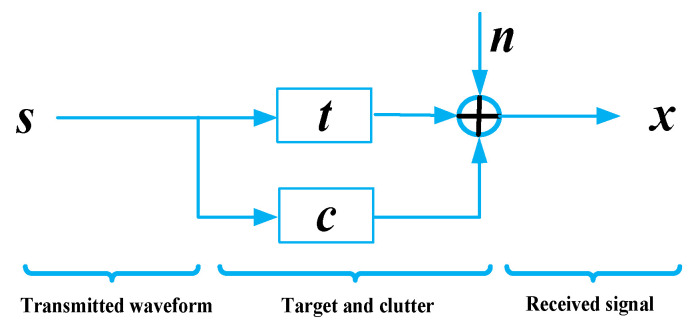
Signal Model.

In this paper, we consider the waveform design of cognitive radar for target estimation in the presence of signal-dependent clutter. The scattering characteristic of the target is represented by the target impulse response (TIR) [28], and the signal-dependent clutter is represented by the clutter impulse response (CIR) [29]. Generally, the prior knowledge of the target and environment (noise and clutter) can be obtained by some cognitive methods [30,31] and is assumed to be known when designing waveform for simplicity. It is assumed that the influence of sidelobes has been mitigated by sidelobe blanking technology in front of the receiver. Meanwhile, we focus on the analysis of single-input single-output radar in this paper which can be straightforwardly extended to multiple-input multiple-output radar case. Then, the discrete baseband signal model is shown in Figure 1.

As illustrated in Figure 1, the target and clutter are modelled by the finite impulse response filter, and the waveform is assumed to be energy-limited. $s\in {\mathbb{C}}^{N_{s}\times 1}$ denotes a transmitted waveform with length $N_{s}$ $t\in {\mathbb{C}}^{N_{t}\times 1}$ and $c\in {\mathbb{C}}^{N_{c}\times 1}$ denote the TIR and CIR, respectively. According to the radar signal model in [8], it is assumed that $N_{t}=N_{c}$ to simplify the derivations. If $N_{c}>N_{t}$, it is necessary to apply a zero-filling operation to the TIR to make the TIR and CIR sampling points equal. $n\in {\mathbb{C}}^{N_{n}\times 1}$ denotes the sum of the noise and the interference, $N_{n}=N_{s}+N_{t}-1$. Let $N=N_{n}$, and ***x*** is the echo with length $N_{x}=N$. Then, the model can be described as:

(1) x =t∗s+c∗s+n=Ts+Cs+n=St+Sc+n

where St=Ts and Sc=Cs can be obtained due to the reciprocity of the convolution operation. The convolution matrices S and T are Toeplitz matrices corresponding to s and t, respectively. We use the function ‘F(·)’ represents their mapping relationship in this paper, i.e., T=F(t), S=F(s). Taking transmitted waveform as an example, the convolution matrix S can be written as:

(2) $$S=\left[\begin{array}{cccc} s\left(1\right) & 0 & \cdots & 0\\ \vdots & s\left(1\right) & \ddots & \vdots \\ s(N_{s}) & \vdots & \ddots & 0\\ 0 & s(N_{s}) & \ddots & s\left(1\right)\\ \vdots & \ddots & \ddots & \vdots \\ 0 & \cdots & 0 & s(N_{s}) \end{array}\right]\in {\mathbb{C}}^{\left(N_{s}+N_{t}-1\right)\times N_{t}}.$$

## 3. Waveform Design Method

In this section, we utilize the MM technique to solve the estimation waveform design problem based on information theory.

### 3.1. MM Method

The MM method refers to the minorization–maximization method, which can transform the original complex problem into a series of simple problems that can be tackled efficiently and converge to the stationary optimal solution of the original problem [22]. Now we first give a brief description of MM. Consider a general maximization problem

(3) $$\begin{array}{l} \underset{x}{\max}\;\;f\left(x\right)\\ s.t.\;x\in \Theta \end{array}$$

where f(x) is a function which is difficult to solve directly. Then, the approximate function $Y\left(x;x^{k}\right)$ is commonly used to replace the original function f(x). More precisely, MM can get the optimal solution $x^{k+1}$ of the (k+1)th iteration based on the known $x^{k}$ according to the following criterion.

(4) $$x^{k+1}\in \underset{x\in \Theta }{\arg\;\max}\;\;Y\left(x;x^{k}\right)$$

where $Y\left(x;x^{k}\right)$ is said to minorize the function f(x) at the point $x^{k}$, which satisfies

(5) $$f\left(x\right)\geq Y\left(x;x^{k}\right)\;\mathrm{for}\;\forall x\in \Theta ,$$

(6) $$f\left(x^{k}\right)=Y\left(x^{k};x^{k}\right)$$

Then, it can be seen that the objective value is increased monotonically at every iteration, i.e.,

(7) $$f\left(x^{k+1}\right)\geq Y\left(x^{k+1};x^{k}\right)\geq Y\left(x^{k};x^{k}\right)=f\left(x^{k}\right)$$

The first inequality and the third equality hold due to the properties of (5) and (6), respectively. The second inequality holds according to (4). Next, MM is utilized to solve the estimation waveform based on information theory.

### 3.2. Problem Formulation

In this paper, the maximization of MI between the received signal and target is used as the optimization criterion for waveform design. According to [28], supposing ***t***, c and n are mutually independent and $t∼CN(0,R_{t})$, $c∼CN(0,R_{c})$, and $n∼CN(0,R_{n})$. Then, the MI of received signal x and target t can be formulated as [32]:

(8) I(x;t|S)=h(x|S)−h(x|t,S)

where h(x|S) denotes the entropy of received signal x when the Toeplitz matrix of transmit waveform S is known, and h(x|t,S) denotes the entropy of x when S and TIR are known.

For the given S, t and x obey the joint Gaussian distribution which can be expressed as:

(9) $$\left(\begin{array}{c} t\\ x \end{array}\right)∼CN\left[\left(\begin{array}{c} 0_{N_{t}}\\ 0_{N_{x}} \end{array}\right),\left(\begin{array}{cc} R_{t} & R_{t}S^{H}\\ SR_{t} & SR_{t}S^{H}+SR_{c}S^{H}+R_{n} \end{array}\right)\right]$$

Let $R_{x}=SR_{t}S^{H}+SR_{c}S^{H}+R_{n}$, we can get

(10) $$p\left(x|S\right)=\frac{1}{\pi^{N_{x}}\det\left(R_{x}\right)}\exp\left[-x^{H}R_{x}^{-1}x\right]$$

Then the entropy h(x|S) and h(x|t,S) can be expressed as:

(11) $$\begin{array}{ll} h\left(x|S\right) & =-\int p\left(x|S\right)\ln p\left(x|S\right)dx\\ & =\ln\det\left(SR_{t}S^{H}+SR_{c}S^{H}+R_{n}\right)+N_{x}\ln\pi +N_{x} \end{array}$$

(12) $$\begin{array}{ll} h\left(x|t,S\right) & =-\int p\left(x|t,S\right)\ln p\left(x|t,S\right)dx\\ & =\ln\det\left(SR_{c}S^{H}+R_{n}\right)+N_{x}\ln\pi +N_{x} \end{array}$$

Bring (11) and (12) into (8), the objective function can be written as:

(13) $$I\left(x,t|s\right)=\ln\det\left[I_{N_{x}}+SR_{t}S^{H}{\left(SR_{c}S^{H}+R_{n}\right)}^{-1}\right]$$

To meet the hardware constraints and maximize the power efficiency, the PAR constraint must be considered. Let the total energy of the transmitted waveform be $E_{s}$. Without any loss of generality, it can be assumed that $E_{s}=N_{s}$. Then, PAR can be defined as:

(14) $$\mathrm{PAR}(s)≜\frac{\max_{j}{\left|s(j)\right|}^{2}}{\frac{1}{N_{s}}{\sum_{n=1}^{N_{s}}\left|s(j)\right|}^{2}}=\underset{j}{\max}{\left|s\left(j\right)\right|}^{2}\leq \eta ,\;\eta \in \left[1,N_{s}\right]$$

where s(j) is the jth element of s, and η is a predefined parameter that denotes the maximum allowed PAR. Note that the PAR constraint is equivalent to a unimodular constraint when η=1, while it becomes a redundant constraint when $\eta =N_{s}$.

Then, the optimization problem can be formulated as:

(15) $$P\left\{ \begin{array}{l} \underset{S}{\max\;}\ln\det\left[I_{N_{x}}+SR_{t}S^{H}{\left(SR_{c}S^{H}+R_{n}\right)}^{-1}\right]\\ s.t.\;\;s^{H}s\leq E_{s}\\ \;\;{\left|s\left(j\right)\right|}^{2}\;\leq \eta ,\;j=1,2,\cdots ,N_{s}\\ \;\;S=F\left(s\right). \end{array}\right.$$

It can be seen that the objective function in problem P is non-convex, the two quadratic inequality constraints are nonhomogeneous [33]. So P is a non-convex problem which is difficult to solve. Therefore, we need to transform P into a convex problem.

### 3.3. Waveform Design

The key to solving P is to convert the non-convex objective function into a convex function. First, the objective function in (13) can be reformulated as:

(16) $$\ln\det\left[I_{N_{x}}+SR_{t}S^{H}{\left(SR_{c}S^{H}+R_{n}\right)}^{-1}\right]=\ln\det\left[I_{N_{t}}+R_{t}^{1/2}S^{H}{\left(SR_{c}S^{H}+R_{n}\right)}^{-1}SR_{t}^{1/2}\right]$$

According to the Woodbury identity [34], we can have

(17) $$\begin{array}{l} \ln\det\left[I_{N_{t}}+R_{t}^{1/2}S^{H}{\left(SR_{c}S^{H}+R_{n}\right)}^{-1}SR_{t}^{1/2}\right]\\ =-\ln\det\left[I_{N_{t}}-R_{t}^{1/2}S^{H}{\left[\left(SR_{c}S^{H}+R_{n}\right)+SR_{t}S^{H}\right]}^{-1}SR_{t}^{1/2}\right]\\ =-\ln\det\left[I_{N_{t}}-R_{t}^{1/2}S^{H}{\left(SR_{\mathrm{tc}}S^{H}+R_{n}\right)}^{-1}SR_{t}^{1/2}\right] \end{array}$$

where $R_{\mathrm{tc}}=R_{t}+R_{c}$. Let $J=\left[\begin{array}{l} I_{N_{t}}\\ 0_{N\times N_{t}} \end{array}\right]\in {\mathbb{C}}^{\left(N_{t}+N\right)\times N_{t}}$ and

(18) $$V=\left[\begin{array}{cc} I_{N_{t}} & R_{t}^{1/2}S^{H}\\ SR_{t}^{1/2} & SR_{tc}^{1/2}S^{H}+R_{n} \end{array}\right]\in {\mathbb{C}}^{\left(N_{t}+N\right)\times \left(N_{t}+N\right)}$$

According to the inversion identity of block matrix [34], the expression in (17) can be reformulated as:

(19) $$I_{N_{t}}-R_{t}^{1/2}S^{H}{\left(SR_{tc}S^{H}+R_{n}\right)}^{-1}SR_{t}^{1/2}={\left(J^{H}V^{-1}J\right)}^{-1}$$

So, we can recast the objective function of P as follows:

(20) $$\ln\;\det\left[I_{N_{t}}+R_{t}^{1/2}S^{H}{\left(SR_{c}S^{H}+R_{n}\right)}^{-1}SR_{t}^{1/2}\right]=\ln\;\det\left[J^{H}V^{-1}J\right]$$

The following lemma provides a way to solve the non-convex design problem by utilizing the MM approach.

**Lemma** **1:** *For any full-column rank matrix*$J\in {\mathbb{C}}^{n\times m}\left(m\leq n\right)$*, if*$V\in {\mathbb{C}}^{n\times n}$*is a positive definite matrix, then*$\ln\;\det\left[J^{H}V^{-1}J\right]$*is convex w.r.t.*V.

Then the proof of Lemma 1 can be found in [35]. Based on Lemma 1, we can find that J is a full-column rank matrix and V is a positive definite matrix, so we can know that $\ln\;\det\left[J^{H}V^{-1}J\right]$ is convex w.r.t. V. Therefore, by using its tangent plane [33] with a given V, this term can be minorized as:

(21) $$\ln\det\left[J^{H}V^{-1}J\right]\geq \ln\det\left[J^{H}{\left(V^{k}\right)}^{-1}J\right]+\mathrm{tr}\left[Q^{\left(k\right)}\left(V-V^{k}\right)\right]$$

where $Q^{k}=-{\left(V^{k}\right)}^{-1}J\left[J^{H}{\left(V^{k}\right)}^{-1}J\right]J^{H}{\left(V^{k}\right)}^{-1}$, the right-hand side of (21) is the first-order approximation of $\ln\det\left[J^{H}V^{-1}J\right]$ for a given $V^{k}$ at kth iteration. Let $Q^{k}=\left[\begin{array}{cc} Q_{11}^{k} & Q_{12}^{k}\\ {\left(Q_{12}^{k}\right)}^{H} & Q_{22}^{k} \end{array}\right]$ with the same partitioning as that of V in (18), where $Q_{11}^{k}\in {\mathbb{C}}^{N_{t}\times N_{t}}$, $Q_{12}^{k}\in {\mathbb{C}}^{N_{t}\times N}$, and $Q_{22}^{k}\in {\mathbb{C}}^{N\times N}$. Now the MM is applied to minorize the function of the left side in (21). Then, the right-hand side of (21) can be rewritten as:

(22) $$Q_{0}^{k}+\mathrm{tr}\left[Q_{12}^{k}SR_{t}^{1/2}\right]+\mathrm{tr}\left[{\left(Q_{12}^{k}\right)}^{H}R_{t}^{1/2}S^{H}\right]+\mathrm{tr}\left[Q_{22}^{k}SR_{tc}S^{H}\right]$$

where $Q_{0}^{k}=\ln\det\left[J^{H}{\left(V^{k}\right)}^{-1}J\right]+\mathrm{tr}\left[Q_{11}^{k}+Q_{22}^{k}R_{n}\right]$ is the constant term.

Ignoring the constant term, and using the identity that tr[AB]=tr[BA] [34], the problem P can be recast as:

(23) $$P_{1}\left\{ \begin{array}{l} \underset{S}{\max\;}\mathrm{tr}\left[SR_{t}^{1/2}Q_{12}^{k}+{\left(Q_{12}^{k}\right)}^{H}R_{t}^{1/2}S^{H}+Q_{22}^{k}SR_{tc}S^{H}\right]\\ s.t.\;s^{H}s\leq E_{s}\\ \;\;{\left|s\left(j\right)\right|}^{2}\;\leq \eta ,\;j=1,2,\cdots ,N_{s}\\ \;\;S=F\left(s\right). \end{array}\right.$$

It can be seen that the objective function of (23) is still non-convex [33]. Then, let $\lambda_{\min}$ denotes the smallest eigenvalue of $SR_{t}^{1/2}Q_{12}^{k}+Q_{21}^{k}R_{t}^{1/2}S^{H}+Q_{22}^{k}SR_{tc}S^{H}$, so the problem $P_{1}$ can be rewritten as:

(24) $$P_{2}\left\{ \begin{array}{l} \underset{S,\lambda_{\min}}{\max\;}\;\lambda_{\min}\\ s.t.\;SR_{t}^{1/2}Q_{12}^{k}+{\left(Q_{12}^{k}\right)}^{H}R_{t}^{1/2}S^{H}+Q_{22}^{k}SR_{tc}S^{H}≽\lambda_{\min}I_{N}\\ \;\;s^{H}s\leq E_{s}\\ \;\;{\left|s\left(j\right)\right|}^{2}\;\leq \eta ,\;j=1,2,\cdots ,N_{s}\\ \;\;S=F\left(s\right). \end{array}\right.$$

Then, the first constraint can be converted to a convex set by utilizing the Schur complement theorem [36] which is defined as follows:

**Lemma** **2.** **(Schur complement theorem):***Let*$A=\left[\begin{array}{cc} A_{11} & A_{12}\\ A_{12}^{H} & A_{22} \end{array}\right]$*, then we can get*A≻0*if and only if*$A_{22}≻0$*and*$A_{11}-A_{12}A_{22}^{-1}A_{12}^{H}≻0$.

According to the Schur complement theorem, the first constraint of this problem is equivalent to

(25) $$\left[\begin{array}{cc} -\lambda_{\min}I_{N}+SR_{t}^{1/2}Q_{12}^{k}+{\left(Q_{12}^{k}\right)}^{H}R_{t}^{1/2}S^{H} & {\left(Q_{22}^{k}\right)}^{1/2}S\\ S^{H}{\left(Q_{22}^{k}\right)}^{1/2} & -R_{tc}^{-1} \end{array}\right]≽0$$

To make the optimization problem more intuitional, $P_{2}$ can be recast as:

(26) $$P_{3}\left\{ \begin{array}{l} \underset{S,\;\lambda_{\min}}{\max\;}\;\lambda_{\min}\\ s.t.\;\left[\begin{array}{cc} -\lambda_{\min}I_{N}+SR_{t}^{1/2}Q_{12}^{k}+{\left(Q_{12}^{k}\right)}^{H}R_{t}^{1/2}S^{H} & {\left(Q_{22}^{k}\right)}^{1/2}S\\ S^{H}{\left(Q_{22}^{k}\right)}^{1/2} & -R_{tc}^{-1} \end{array}\right]≽0\\ \;\;{\left[S(m:m+N_{s}-1,m)\right]}^{H}S(m:m+N_{s}-1,m)\leq E_{s},\;m=1,2,\cdots ,N_{t}\\ \;\;{\left|S\left(i,j\right)\right|}^{2}\;\leq \eta ,\;i=1,2,\cdots ,N,\;\;\;j=1,2,\cdots ,N_{t}. \end{array}\right.$$

where ‘$S(m:m+N_{s}-1,m)$’ represents the elements in the mth column, and the mth to $\left(m+N_{s}-1\right)\mathrm{th}$ rows of S. We can see that the first inequality constraint is the linear inequality with regard to matrix variable S. According to [33], any line is affine, so the first inequality constraint is a convex set. Then the second constraint belongs to a Euclidean ball, and the third constraint belongs to a norm ball, and both of them are convex set [33]. Therefore, the constraints of $P_{3}$ are convex sets with regard to matrix S.

In addition, it is obvious that the objective function of $P_{3}$ is an auxiliary variable which is also convex. Hence, $P_{3}$ is a convex problem with regard to matrix variable S and it can be solved by applying the interior point method [33] with CVX toolbox [37]. However, it has an approximate computational complexity of $O\left(N_{s}^{3.5}\right)$ [38] at each iteration, which may bring a high computation cost especially when $N_{s}$ is large. Therefore, a fast optimization method is needed.

### 3.4. A Fast Optimization Method

To reduce the cost of computation, we should convert the original problem $P_{1}$ to a form that is easier to solve. First, we can convert the matrix variable S to the form of vector. Then, the identities that $\mathrm{tr}\left(AB\right)=\mathrm{ve}c^{T}\left(B^{T}\right)\mathrm{vec}\left(A\right)$ and $\mathrm{tr}\left(ABCD\right)=\mathrm{ve}c^{T}\left(D^{T}\right)\left(C^{T}⊗A\right)\mathrm{vec}\left(B\right)$ [39] can be used to recast the objective function in (23), which can be rewritten as:

(27) $$\begin{array}{l} \mathrm{tr}\left[SR_{t}^{1/2}Q_{12}^{k}+{\left(Q_{12}^{k}\right)}^{H}R_{t}^{1/2}S^{H}+Q_{22}^{k}SR_{tc}S^{H}\right]\\ =\mathrm{ve}c^{T}\left({\left(R_{t}^{1/2}Q_{12}^{k}\right)}^{T}\right)\mathrm{vec}\left(S\right)+\mathrm{ve}c^{T}\left({\left(S^{H}\right)}^{T}\right)\mathrm{vec}\left({\left(Q_{12}^{k}\right)}^{H}R_{t}^{1/2}\right)+\mathrm{ve}c^{T}\left({\left(S^{H}\right)}^{T}\right)\left(R_{tc}^{T}⊗Q_{22}^{k}\right)\mathrm{vec}\left(S\right)\\ ={\left(u^{k}\right)}^{T}\mathrm{vec}\left(S\right)+\mathrm{ve}c^{T}\left({\left(S^{H}\right)}^{T}\right)v^{k}+\mathrm{ve}c^{T}\left({\left(S^{H}\right)}^{T}\right)G^{k}\mathrm{vec}\left(S\right) \end{array}$$

where

(28) $$\begin{array}{l} u^{k}=\mathrm{vec}\left({\left(R_{t}^{1/2}Q_{12}^{k}\right)}^{T}\right)\in {\mathbb{C}}^{N_{t}N\times 1},\\ v^{k}=\mathrm{vec}\left({\left(Q_{12}^{k}\right)}^{H}R_{t}^{1/2}\right)\in {\mathbb{C}}^{N_{t}N\times 1},\\ G^{k}=\left(R_{tc}^{T}⊗Q_{22}^{k}\right)\in {\mathbb{C}}^{N_{t}N\times N_{t}N} \end{array}$$

For further simplification, considering that S is a convolution matrix with Toeplitz structure (shown in (2)) which consists of s, we can recast the right hand side of (27) as:

(29) $${\left({\tilde{u}}^{k}\right)}^{T}s+s^{H}{\tilde{v}}^{k}+s^{H}{\tilde{G}}^{k}s$$

where

(30) $$\begin{array}{l} {\tilde{u}}^{k}=\sum_{i=1}^{N_{t}}{\tilde{u}}_{i}^{k}\in {\mathbb{C}}^{N_{s}\times 1},\\ {\tilde{u}}_{i}^{k}=\left[u^{k}\left(\left(i-1\right)*N+i\right),u^{k}\left(\left(i-1\right)*N+i+1\right),\cdots ,u^{k}\left(\left(i-1\right)*N+i+N_{s}-1\right)\right] \end{array}$$

(31) $$\begin{array}{l} {\tilde{v}}^{k}=\sum_{i=1}^{N_{t}}{\tilde{v}}_{i}^{k}\in {\mathbb{C}}^{N_{s}\times 1},\\ {\tilde{v}}_{i}^{k}=\left[v^{k}\left(\left(i-1\right)*N+i\right),v^{k}\left(\left(i-1\right)*N+i+1\right),\cdots ,v^{k}\left(\left(i-1\right)*N+i+N_{s}-1\right)\right] \end{array}$$

and ${\tilde{G}}^{\left(k\right)}=\sum_{i=1}^{N_{t}}\sum_{j=1}^{N_{t}}B_{i,j}^{k}$, where $B_{i,j}^{k}\in {\mathbb{C}}^{N_{s}\times N_{s}}$ can be expressed as:

(32) $$B_{i,\;j}^{k}=\left[\begin{array}{ccc} G^{k}\left[\left(i-1\right)N+j\;,\left(\;i-1\right)N+j\right] & \cdots & G^{k}\left[\left(i-1\right)N+j\;,\left(\;i-1\right)N+j+N_{s}-1\right]\\ \vdots & \ddots & \vdots \\ G^{k}\left[\left(i-1\right)N+j+N_{s}-1\;,\left(\;i-1\right)N+j\right] & \cdots & G^{k}\left[\left(i-1\right)N+j+N_{s}-1\;,\left(\;i-1\right)N+j+N_{s}-1\right] \end{array}\right]$$

Then the optimization problem $P_{1}$ can be further rewritten as:

(33) P4{max sRe(sHrk)+sHG˜kss.t. sHs≤Es  |s(j)|2 ≤η, j=1,2,⋯,Ns.

where $r^{k}={\tilde{u}}^{k}+{\tilde{v}}^{k}$. It can be seen that $P_{4}$ is a quadratically constrained quadratic program (QCQP) problem, which can be solved by the power method-like in [26]. More precisely, $P_{4}$ can be reformulated as:

(34) $$P_{5}\left\{ \begin{array}{l} \underset{s}{\min\;}{\left\|s-a^{k}\right\|}_{2}^{2}\\ s.t.\;s^{H}s\leq E_{s}\\ \;\;{\left|s\left(j\right)\right|}^{2}\;\leq \eta ,\;j=1,2,\cdots ,N_{s} \end{array}\right.$$

where $a^{k}$ consists of the first $N_{s}$ entries of ${\tilde{W}}^{k}{\tilde{s}}^{k}$, ${\tilde{s}}^{k}={\left[{\left(s^{k}\right)}^{T},\;1\right]}^{T}$, let

(35) $$W^{k}=\left[\begin{array}{cc} {\tilde{G}}^{k} & r^{k}\\ {\left(r^{k}\right)}^{H} & 0 \end{array}\right]$$

Then ${\tilde{W}}^{k}=\mu^{k}I_{N_{s}+1}-W^{k}$, where $\mu^{k}$ is a constant that is larger than the maximum eigenvalue of $W^{k}$ to make sure that ${\tilde{W}}^{k}$ is positive definite. It can be seen that $P_{5}$ is a convex problem which can be solved by using the interior point method. However, we can find that the form of objective function and constraints of $P_{5}$ are the same as the nearest neighbor method with a lower complexity of $O\left(N_{s}^{2}\right)$ [40]. Hence, $P_{5}$ can be solved efficiently.

Then, let $I^{k}$ denote the value of MI at kth iteration, τ the termination tolerance and γ the maximum iterative number. According to the above steps, the proposed MM-based method is summarized in Box 1.

Box 1 **The proposed minimization–maximization (MM)-based method for low-PAR estimation waveform design.**

**Step** **0:** Set k=0, generate a random waveform $s^{k}$, initialize the τ and γ.
**Step** **1:** Use (28) to update $u^{k}$, $v^{k}$ and $G^{k}$;
**Step** **2:** Use (30) and (31) to update ${\tilde{u}}^{k}$ and ${\tilde{v}}^{k}$;
**Step** **3:** Use (32) to update $B_{i,j}^{k}$, ${\tilde{G}}^{k}=\sum_{i=1}^{N_{t}}\sum_{i=1}^{N_{t}}B_{i,j}^{k}$;
**Step** **4:** $r^{k}={\tilde{u}}^{k}+{\tilde{v}}^{k}$, use (35) to update $W^{k}$, ${\tilde{W}}^{k}=\mu^{k}I_{N_{s}+1}-W^{k}$;
**Step** **5:** Get $a^{k}$ from the first $N_{s}$ entries of ${\tilde{W}}^{k}{\tilde{s}}^{k}$;
**Step** **6:** Solve $P_{5}$ to update $s^{k+1}$, set k=k+1;
**Step** **7:** Go back to step 1 until $\left|I^{k}-I^{k-1}\right|/I^{k}\leq \tau$ or the iteration number is larger than γ.

### 3.5. Acceleration Scheme

For the MM method, the convergence speed depends mainly on the minorized function. It is worth noting that the minorized function of the proposed method (as shown in the right-hand side of (21)) may be relatively loose as a lower bound of the original function. Therefore, although the computational cost of the proposed method is low at each iteration, the convergence speed may still be slow. For the purpose of accelerating the convergence speed, the acceleration scheme (SQUAREM) in [41] is adopted in this paper. Then, we give the modified version of SQUAREM according to the optimization problem we meet.

Let $L_{\mathrm{MM}}\left(\cdot \right)$ denote the fixed-point map of the proposed MM-based method which can be described as:

(36) $$s^{k+1}=L_{\mathrm{MM}}\left(s^{k}\right)$$

However, SQUAREM is not applicable to the case of the limited-energy and PAR constraints, and the monotonicity of the proposed method cannot be guaranteed by using SQUAREM. To this end, the first problem can be solved by using the nearest neighbor method to deal with $P_{5}$, and the second problem can be tackled by utilizing a backtracking strategy. Then, the acceleration scheme based on SQUAREM is summarized in Box 2.

Box 2 **The Acceleration scheme based on squared iterative methods (SQUAREM).**

**Step** **0:** Set *k* = 0, generate a random waveform ***s**^k^*, initialize the *τ* and *γ*;
**Step** **1:** $s_{1}=L_{\mathrm{MM}}\left(s^{k}\right)$;
**Step** **2:** $s_{2}=L_{\mathrm{MM}}\left(s_{1}\right)$;
**Step** **3:** $e=s_{1}-s^{k}$;
**Step** **4:** $q=s_{2}-s_{1}-e$;
**Step** **5:** α=−‖e‖/‖q‖;
**Step** **6:** $a^{k}=s^{k}-2\alpha e+\alpha^{2}q$;
**Step** **7:** Solve to ***P***_5_ update ***s***^*k*+1^;
**Step** **8:** **while**$I^{k+1}<I^{k}$ do
**Step** **9:**   α=(α−1)/2;
**Step** **10:**  $a^{k}=s^{k}-2\alpha e+\alpha^{2}q$;
**Step** **11:**  Solve ***P***_5_ to update ***s***^*k*+1^;
**Step** **12:** **end while**
**Step** **13:** Set *k* = *k* + 1;
**Step** **14:** Go back to step 1 until $\left|I^{k}-I^{k-1}\right|/I^{k}\leq \tau$ or the iteration number is larger than *γ*.

## 4. Performance Analysis

### 4.1. Convergence

Let $U\left(s;s^{k}\right)$ represent the minorized function (i.e., the objective function of $P_{4}$) which can be written as:

(37) U(s;sk)=Re(sHrk)+sHG˜ks

As is well known, $P_{4}$ is a QCQP problem. According to [42], it had been proved that $U\left(s^{k+1};s^{k}\right)>U\left(s^{k};s^{k}\right)$ under the PAR and transmitted energy constraints. Then, we can have

(38) $$I\left(s^{k}\right)=U\left(s^{k};s^{k}\right)\leq U\left(s^{k+1};s^{k}\right)\leq I\left(s^{k+1}\right)$$

where $I\left(s^{k}\right)$ denotes MI value of the original function at kth iteration. The first equality and the third inequality hold due to the properties of (6) and (5), respectively. Hence, we can know that the proposed method is monotonically increasing.

In addition, the waveform $\left\{s^{k}\right\}$ is energy-limited and its every point bounded with $\left|s^{k}\left(j\right)\right|\;\leq \sqrt{\eta }\;\;\left(j=1,2,\cdots ,N_{s}\right)\;$. Therefore, according to the Theorem 2.17 in [42] and the fact that I(s) and $U\left(s;s^{k}\right)$ have the same gradient value when $s=s^{k}$, we can know that at least one limit point exists and the MI of the synthesized waveform has its upper bound.

### 4.2. Computational Complexity

The proposed MM-based method converts the original problem into a simple problem which can be solved efficiently. In every iteration, updating $Q^{k}$, performing eigenvalue decomposition to obtain $\mu^{k}$, and solving $P_{5}$ with the nearest neighbor method, which have complexities of $O\left(N_{s}^{2}\right)$, $O\left(N_{s}^{3}\right)$ and $O\left(N_{s}^{2}\right)$, respectively. Therefore, the total computation complexity of the proposed method is $O\left(N_{s}^{3}+2N_{s}^{2}\right)$ in every iteration.

## 5. Simulation Results

In this section, several numerical simulations are performed to demonstrate the performance of the proposed method. Assuming that the length of the transmitted waveform is $N_{s}=20$, the initial waveform $s_{0}$ is generated by a random phase-coded signal. The length of TIR and CIR are $N_{t}=N_{c}=30$. Meanwhile, both the target and clutter are mutually independent circular symmetric complex Gaussian random vector, i.e., $t∼CN(0,R_{t})$, $c∼CN(0,R_{c})$ [28], where $R_{t}=\sigma_{t}^{2}R_{t0}$ and $R_{c}=\sigma_{c}^{2}R_{c0}$. According to [5], $R_{t0}=U_{t}\Lambda_{t}U_{t}^{H}$ and $R_{c0}=U_{c}\Lambda_{c}U_{c}^{H}$ are normalized covariance matrix, where $\Lambda_{t}$ and $\Lambda_{c}$ have the same structure. Hence, taking the $\Lambda_{t}$ as an example, $\Lambda_{t}\in {\mathbb{C}}^{N_{t}\times N_{t}}$ is a diagonal matrix and $U_{t}$ is the $N_{t}\times N_{t}$ unitary discrete Fourier transform (DFT) matrix with its (i,j)th entry given by

(39) $$\frac{1}{\sqrt{N_{t}}}\exp\left[\frac{-j2\pi \left(i-1\right)\left(j-1\right)}{N_{t}}\right],\forall i,j\in \left[1,N_{t}\right]$$

The noise is white Gaussian with zero mean and covariance matrix $R_{n}=\sigma_{n}^{2}I_{N}$, where $\sigma_{n}^{2}=0.5$ denotes the variance of noise. Then, we perform 300 Monte Carlo trials for each combination of parameters and the termination tolerance $\tau =10^{-6}$. The MATLAB 2013b version is used to perform the simulations with a standard PC (CPU Core i5-3230M 2.6GHz and 4GB RAM).

### 5.1. Effectiveness Verification

**Figure 2:**
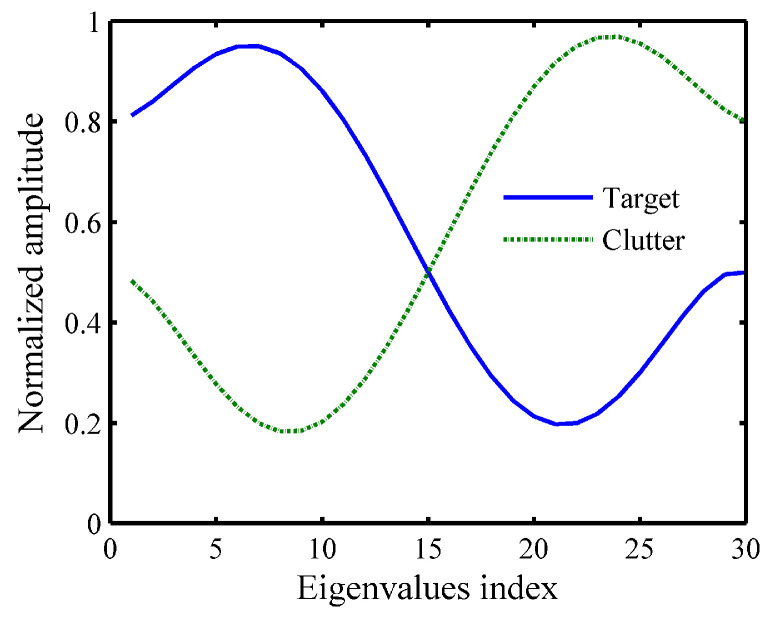
Eigenvalues of the matrices $R_{t0}$ and $R_{c0}$.

In this subsection, we demonstrate the effectiveness of the proposed method. First, we give the typical set of eigenvalues for the normalized matrices $R_{t0}$ and $R_{c0}$ as shown in Figure 2.

**Figure 3:**
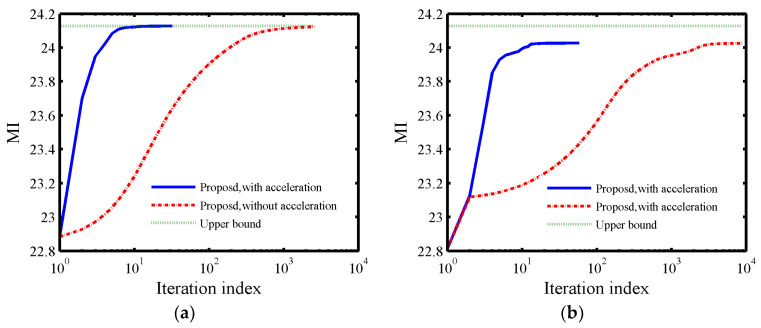
The convergence of the proposed method, (**a**) $\eta =N_{s}$ and (**b**) η=1.

Let the total energy of waveform $E_{s}=N_{s}$, $\sigma_{t}^{2}=1$ and $\sigma_{c}^{2}=1$. Figure 3 shows the convergence of the proposed MM-based method. In addition, the upper bound is obtained by using the Lagrange multipliers method to solve $P_{4}$ under only the energy constraint. From Figure 3a ($\eta =N_{s}$ which is equivalent to the energy constraint) and Figure 3b (η=1 which is equivalent to the constant-modulus constraint), it can be seen that the accelerated case is much faster than the case without acceleration. Meanwhile, the MI of the synthesized waveform with $\eta =N_{s}$ can get the upper bound and the case with η=1 is about 0.07 away from the upper bound, this is because the large PAR value ($\eta =N_{s}$) has larger feasible set region than small PAR value (η=1).

**Figure 4:**
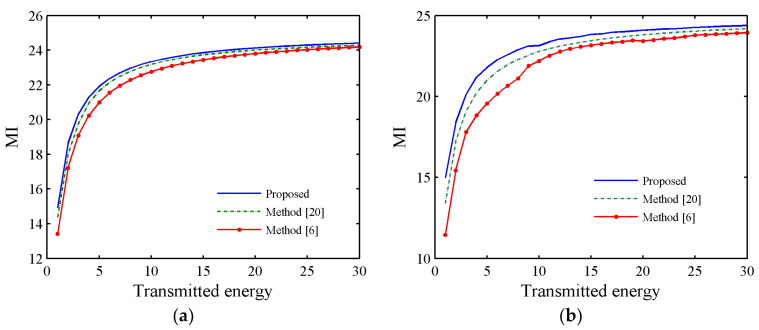
Estimation performance of different waveforms versus transmitted energy, (**a**) $\eta =N_{s}$, (**b**) $\eta ={E_{s}/N}_{s}$.

Let the transmitted energy $E_{s}$ range from 1 to 30, $\sigma_{t}^{2}=1$ and $\sigma_{c}^{2}=1$. Then, Figure 4a,b shows the estimation performance comparison of the proposed method, the Sequence Linear Programming-based Waveform Design algorithm (SLPWD) in [20] and the frequency domain-based Cognitive REceiver and Waveform design algorithm (CREW(fre)) in [6] versus the transmitted energy with $\eta =E_{s}$ and $\eta ={E_{s}/N}_{s}$ (i.e., constant-modulus constraint), respectively.

From Figure 4 we can see that the MI of proposed method is larger than that of other methods. Meanwhile, the MI of SLPWD algorithm in [20] is larger than CREW(fre) algorithm in [6], this is because CREW(fre) addresses the waveform design in frequency domain and the waveform spectrum does not contain the phase information, which may result in a decline of the waveform performance when the waveform spectrum is converted to time domain.

**Figure 5:**
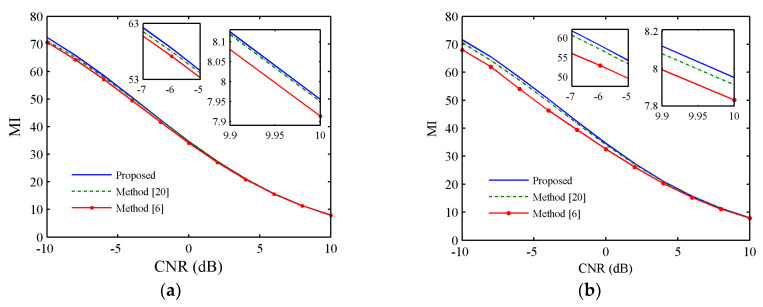
Estimation performance comparison of different waveforms versus clutter-noise-ration (CNR), (**a**) $\eta =E_{s}$, (**b**) η=1.

Let $E_{s}=N_{s}$, $\sigma_{t}^{2}=1$, the clutter-noise-ration (CNR) ranges from −10 dB to 10 dB. Figure 5a,b shows the estimation performance of the proposed method, SLPWD in [20] and CREW(fre) in [6] versus CNR with $\eta =E_{s}$ and η=1, respectively. It can be seen that the MI of the proposed method is better than the other methods. Hence, it demonstrates the effectiveness of the proposed method.

**Figure 6:**
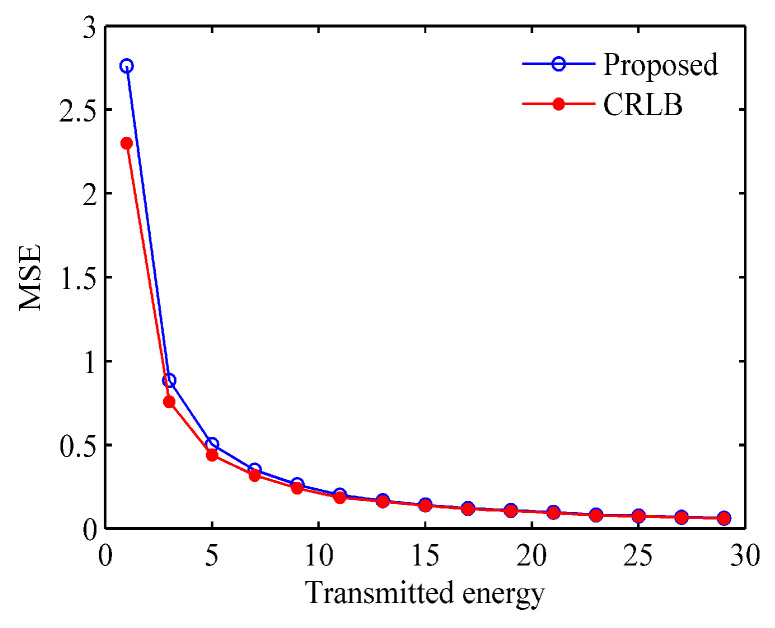
The estimation performance assessment of the optimal waveform.

Figure 6 shows the performance assessment for the estimation of target t with the proposed method. For the optimal waveform based on maximizing MI, mean squared error (MSE) will be used for performance assessment. In addition, we also compared with the CRLB. The MSE and CRLB for transmitted waveform are derived in Appendix A. From Figure 6 we can see that the estimated performance gets closer to the CRLB as the transmitted energy increases. So, it verifies that the waveform generated by the proposed method has good estimation performance.

### 5.2. Influence of PAR

**Figure 7:**
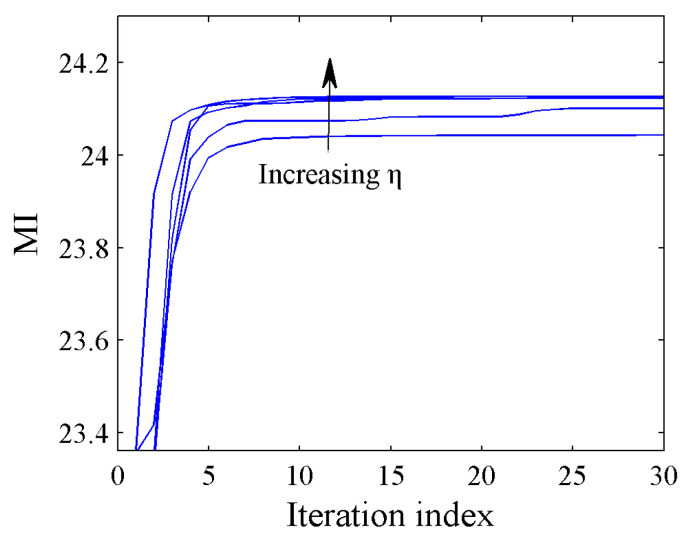
Comparison of waveforms under different peak-to-average power ratio (PAR) constraints $\eta =[1,1.2,1.5,2,3,N_{s}]$.

**Figure 8:**
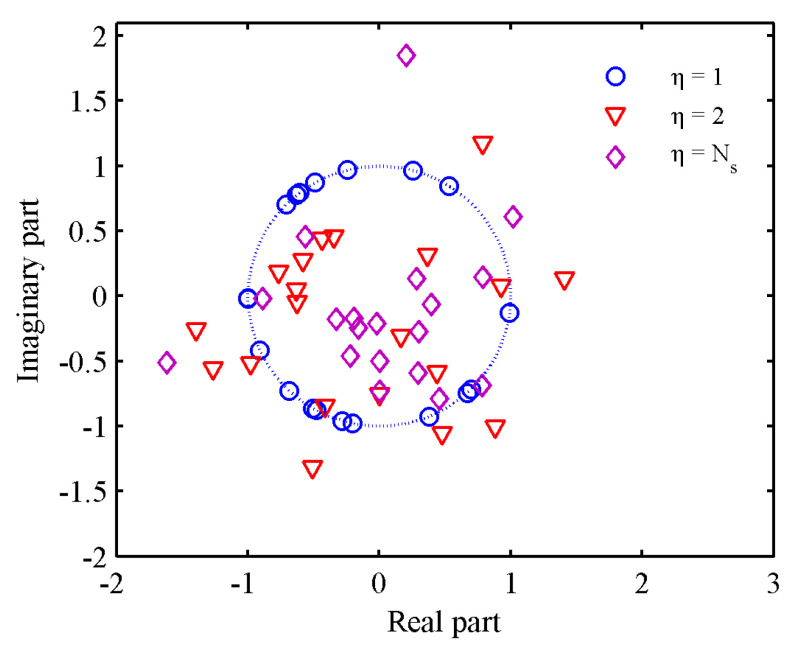
Real and imaginary parts of waveforms with $\eta =[1,2,N_{s}]$.

In this subsection, we discuss the influence of the PAR on the synthesized waveform. Figure 7 shows the MI of the synthesized waveforms under different PAR values. We can see that the curves can converge to their respective stationary values which become larger as η increases, and this is because the feasible set region in $P_{5}$ becomes larger as η increases. However, since the energy of the transmitted waveform is limited, the waveform performance has its upper bound. Then, we can also see that the curves can be monotonically convergent to the same stationary value and the curves almost overlap when η≥1.5. Figure 8 shows the real and imaginary parts of the waveforms under different PAR constraints. When $\eta =N_{s}$, the distribution radii of the corresponding points are large, which is not favorable for practical applications. In contrast, the results obtained with η=1 are unimodular and lie on the unit circle. Meanwhile, the distribution radii of the waveform with η=2 are close to those of the waveform with η=1, and the performance is very close to that of the waveform with $\eta =N_{s}$, as shown in Figure 7. This result indicates that the low-PAR waveform (for example η=2) not only meet the hardware constraints but also have better estimation performance than a unimodular waveform. Hence, the low-PAR waveform is more suitable for practical applications.

## 6. Conclusions

In this paper, we proposed an efficient low-PAR waveform design method of cognitive radar for the extended target estimation in the presence of signal-dependent clutter. To tackle the original non-convex problem in the time domain, an efficient method is proposed by using the MM technique. Meanwhile, to improve the convergence speed, an acceleration approach is given based on the SQUAREM. Numerical experiments demonstrate the effectiveness of the proposed method for the given PAR. Compared with the existing method, the proposed method demonstrates the advantage w.r.t. estimation performance. Moreover, the proposed method can be used in the waveform design of cognitive radar systems since the high computational efficiency will enable real-time waveform changes. Possible future research tracks include the extension to cases with low autocorrelation sidelobes and spectral constraints with imprecise prior information.

## Appendix A

**MSE and CRLB for the transmitted waveform.** Let $R_{w}=SR_{c}S^{H}+R_{n}$. According to the reference [43], (p. 156), for the given ***x*** and ***S***, the estimation of ***t*** can be written as:

(A1) $$\hat{t}=R_{t}S^{H}{\left(SR_{t}S^{H}+R_{w}\right)}^{-1}x$$

Then the estimation error for ***t*** can be written as:

(A2) $$\begin{array}{ll} \mathrm{MSE} & =E\left\{{\left\|t-R_{t}S^{H}{\left(SR_{t}S^{H}+R_{w}\right)}^{-1}x\right\|}_{2}^{2}\right\}\\ & =\mathrm{tr}\left(R_{t}\right)-2\mathrm{tr}\left[R_{t}S^{H}{\left(SR_{t}S^{H}+R_{w}\right)}^{-1}SR_{t}\right]+\\ & \;\mathrm{tr}\left[R_{t}S^{H}{\left(SR_{t}S^{H}+R_{w}\right)}^{-1}SR_{t}\right]\\ & =\mathrm{tr}\left(R_{t}\right)-\mathrm{tr}\left[R_{t}S^{H}{\left(SR_{t}S^{H}+R_{w}\right)}^{-1}SR_{t}\right] \end{array}$$

For the given ***S***, the likelihood function of ***t*** can be written as:

(A3) $$f(x|t)=\frac{1}{\pi^{N_{x}}\det(R_{w})}\exp\left[-{\left(x-St\right)}^{H}R_{w}^{-1}(x-St)\right]$$

The logarithmic form of (42) can be expressed as:

(A4) $$\ln f\left(x|t\right)=-N_{x}\ln\pi -\ln\det\left(R_{w}\right)-{\left(x-St\right)}^{H}R_{w}^{-1}(x-St)$$

Differentiating objective function in (8) with respect to ***t***, we can have

(A5) $$\frac{\partial \ln f\left(x|t\right)}{\partial t}=2S^{H}R_{w}^{-1}x-2S^{H}R_{w}^{-1}St$$

Then the error matrix can be expressed as:

(A6) $$C_{\mathrm{CRLB}}=\frac{1}{-E\left\{\frac{\partial }{\partial t}{\left[\frac{\partial \ln\;f\left(x|t\right)}{\partial t}\right]}^{T}\right\}}={\left(2S^{H}R_{w}^{-1}S\right)}^{-1}$$

So, the CRLB can be written as:

(A7) $${\mathrm{Err}}_{\mathrm{CRLB}}=\mathrm{tr}\left[{\left(2S^{H}R_{w}^{-1}S\right)}^{-1}\right]$$
